# The characteristics, implementation and effects of Aboriginal and Torres Strait Islander health promotion tools: a systematic literature search

**DOI:** 10.1186/1471-2458-14-712

**Published:** 2014-07-11

**Authors:** Janya McCalman, Komla Tsey, Roxanne Bainbridge, Kevin Rowley, Nikki Percival, Lynette O’Donoghue, Jenny Brands, Mary Whiteside, Jenni Judd

**Affiliations:** 1 The Cairns Institute, James Cook University, McGregor Rd, Smithfield, PO Box 6811, 4870, QLD 4878, Cairns, Australia; 2 Onemda VicHealth Koori Health Unit and Centre for Health and Society, Melbourne School of Population and Global Health, The University of Melbourne, Level 4, 207 Bouverie Street, 3010 Melbourne, VIC, Australia; 3 Menzies School of Health Research, Level 1, 147 Wharf Street, 4000 Spring Hill, QLD, Australia; 4 Menzies School of Health Research, PO Box 41096, 0811 Casuarina, NT, Australia; 5 Department of Social Work & Social Policy, School of Allied Health, Faculty of Health Sciences, La Trobe University, 3068 Bundoora, VIC, Australia; 6 Faculty of Medicine, Health and Molecular Sciences, Anton Brienl Research Centre for Health Systems Strengthening, James Cook University, Townsville, QLD 4811, Australia

**Keywords:** Health promotion, Indigenous, Tools, Guides, Instruments, Packages, Frameworks, Resources, Implementation, Evaluation

## Abstract

**Background:**

Health promotion by and with Aboriginal and Torres Strait Islander (hereafter Indigenous) Australians is critically important given a wide gap in health parity compared to other Australians. The development and implementation of step-by-step guides, instruments, packages, frameworks or resources has provided a feasible and low-resource strategy for strengthening evidence-informed health promotion practice. Yet there has been little assessment of where and how these tools are implemented or their effectiveness. This paper reviews the characteristics, implementation and effects of Indigenous health promotion tools.

**Methods:**

Indigenous health promotion tools were identified through a systematic literature search including a prior scoping study, eight databases, references of other reviews and the authors’ knowledge (n = 1494). Documents in the peer reviewed and grey literature were included if they described or evaluated tools designed, recommended or used for strengthening Indigenous Australian health promotion. Eligible publications were entered into an Excel spreadsheet and documented tools classified according to their characteristics, implementation and effects. Quality was appraised using the Dictionary for Effective Public Health Practice Project (EPHPP) and Critical Appraisal Skills Program (CASP) tools for quantitative and qualitative studies respectively.

**Results:**

The review found that Indigenous health promotion tools were widely available. Of 74 publications that met inclusion criteria, sixty (81%) documented tools developed specifically for the Indigenous Australian population. All tools had been developed in reference to evidence; but only 22/74 (30%) publications specified intended or actual implementation, and only 11/74 (15%) publications evaluated impacts of the implemented tools. Impacts included health, environmental, community, organisational and health care improvements. The quality of impact evaluations was strong for only five (7%) studies.

**Conclusions:**

The small number and generally moderate quality of implementation and evaluation studies means that little is known about how tools work to strengthen Indigenous health promotion practice. The findings suggest that rather than continuing to invest in tool development, practitioners, policy makers and researchers could evaluate the implementation and effects of existing tools and publish the results. There is a need for long-term investment in research to review the current use of health promotion tools and the factors that are likely to enhance their implementation.

## Background

There is considerable scope for improvements in the implementation of health promotion efforts targeting Aboriginal and Torres Strait Islander (hereafter Indigenous) individuals, families, organisations and communities. Health promotion, defined as the process of enabling people to increase control over the determinants of health and thereby improve their health [1], is critically important for Indigenous Australian health improvement [2]. Health promotion can contribute to reducing the greater Indigenous burden of illness and mortality compared to other Australians, particularly as the young age structure of the population means that there is a greater scope for reducing preventable illnesses and conditions. As well, health promotion can contribute to health equity and social justice through empowering responses to the considerable social disadvantage associated with the historical dispossession and contemporary structural and social determinants such as poverty and powerlessness. Improvements in the quality of health promotion skills, organisational structures, resources and commitment to improvement in health and other sectors have the potential to contribute to a sustained increase in health gains many times over [3].

Indigenous perceptions of health recognise a holistic view encompassing physical, mental and spiritual health as well as individual and community levels. Pat Anderson, the Chairperson of Australia’s Indigenous health research centre, the Lowitja Institute, recently argued that the breadth of health promotion efforts needed to be expanded. She stated: “attempts to prevent physical health issues are not enough from our perspective – prevention needs to operate across all these other domains as well. Our holistic conception of health is powerfully supported by the theory of the social determinants of health” [4]. In 2002, a consensus statement by key health promotion representatives from all Australian states and territories acknowledged that Indigenous health issues are complex and health improvement requires inter-sectoral strategies [5].

The demand for use of proven evidence-based strategies to improve Indigenous health has led government and non-government organisations to invest considerably in the development and implementation of tools. Health promotion tools were defined as structured step-by-step guides, instruments, packages, frameworks or resources which are designed for enabling practitioners and organisations to plan, implement or evaluate a health program or improve an existing one. Tools include guidelines, practice models or frameworks, training packages/manuals, toolkits, resource kits, action packs, screening tools, audit tools, handbooks, measurement tools, checklists and networks. Given the limited availability and often short-term nature of health promotion grant funding, the development and implementation of such tools has provided a feasible and low-resource strategy for strengthening Indigenous health promotion approaches, and the internet provides an easily accessible and time-efficient means of disseminating the developed tools. Yet there has been little assessment of the characteristics of such tools, whether or how they meet the priorities and needs of Indigenous Australian contexts, how they are implemented to improve Indigenous health promotion practice and outcomes, and whether they assist in supporting improved health promotion practice or outcomes.

In order to optimise the potential of tools to contribute to Indigenous health promotion, it is important to develop an understanding of how to effectively deliver promising or proven tools across the wide diversity of Indigenous health promotion settings [6]. With these concerns in mind, the Lowitja Institute identified implementation research in Indigenous health promotion as a key priority. A project was established in 2011 by Lowitja’s Healthy Communities and Settings Program through collaborative workshops by researchers and community members to facilitate research development [7,8]. The project aimed to improve knowledge and understanding of the uptake and implementation of tools to strengthen Indigenous health promotion in primary health care and other settings. A scoping study of Indigenous health promotion tools conducted as the first stage of this project [9] identified 93 policy frameworks, project reports, data sources, principles and learnings to inform Indigenous health promotion, but it was difficult to ascertain which tools could be used directly and pragmatically to inform effective health promotion initiatives [10]. This review builds on the earlier scoping study to examine evaluation or program description studies to determine: 1) what are the characteristics of tools designed to promote Indigenous Australian health; 2) how and where have tools been developed and implemented (including the evidence or form of knowledge that was implemented through the tools, the contexts within which tools were implemented, and the methods of facilitation used); and 3) what were the effects of tool implementation on Indigenous health promotion improvement?

## Methods

Publications were identified and classified using a process that was consistent with Cochrane methods for systematic searches [11]. First a protocol for this review (Additional file 1) was circulated to the study co-authors until consensus was reached about the research questions and methods.

### Inclusion criteria

Documents were included in the review if they described or evaluated Indigenous-specific or non-Indigenous specific tools (structured step-by-step guides, instruments, packages, frameworks or resources) that were designed, recommended or used to plan, implement or evaluate an Indigenous Australian health promotion program. The potential of non-Indigenous specific tools to contribute to Indigenous Australian health promotion was determined by: 1) the inclusion of the tool in Wise et al.’s [9] study; 2) recommendation for use in Indigenous Australian health in other reviews; or 3) knowledge by an author of this paper that the tool had been used in Indigenous health. The tools themselves and studies of tools were both included. The time period for the analysis was 2002 to 2012 – a decade of tool development was considered sufficient for analysing the majority of Indigenous health promotion tools and was feasible within the scope of this project. Publications were included only if they were in English. Peer-reviewed and grey literature were included since a substantial proportion of Indigenous health research is published in the grey literature [12]. In cases where a relevant study was published in both the peer-reviewed and grey literature, peer-reviewed publications were prioritised and grey literature included only if it referred to a discrete aspect of a tool not included in its peer reviewed counterpart.

### Search strategy

**Figure 1:**
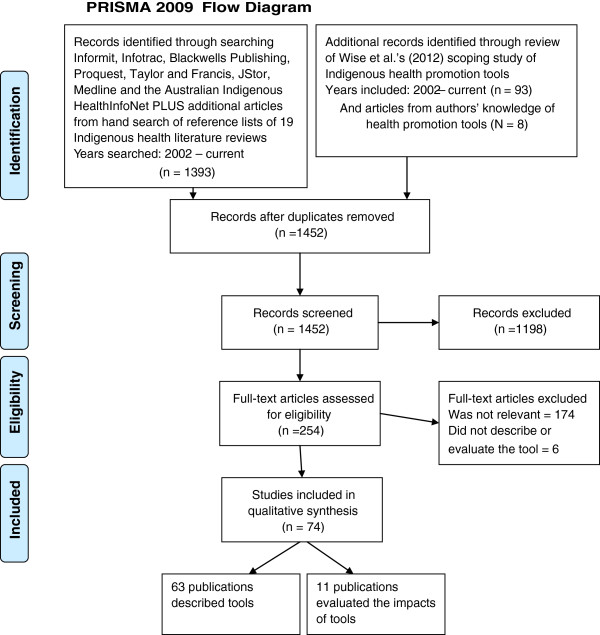
PRISMA 2009 flow diagram.

A search strategy, summarised in Figure 1 (detailed information attached as Additional file 2), was utilised. First, the Indigenous health promotion tools identified in Wise et al.’s [9] earlier scoping study were reviewed to determine whether they met our definition of a tool (n = 93). Second, full-text publications in the peer-reviewed and grey literature were searched through eight electronic databases: Informit, Infotrac, Blackwells Publishing, Proquest, Taylor and Francis, JStor, Medline and the Australian Indigenous HealthInfoNet. The search string for Informit included the following terms in abstracts: Aborigin* OR Indigen* OR Torres AND health AND service OR program* OR intervention OR tool AND Australia (n = 902) (last date: 25 November 2013). As well, the reference lists of 19 Indigenous health-related literature reviews were manually searched (n = 1393) (last date 29 November 2013). Third, the authors of this study drew on their knowledge of health promotion tools (n = 8) (last date 16 May 2013).

A keyword search of the 1494 publications was conducted using the terms: tool*, training*, resource*, guide*, instrument*, package*, framework*, model*, manual*, toolkit*, kit*, pack*, handbook*, checklist* or network*. Publications were excluded that: 1) did not meet our definition of a health promotion tool (n = 1198); 2) were not relevant to Indigenous Australian health promotion (n = 174); 3) did not describe or evaluate the tool (n = 6); and 4) were duplicates (n = 42). Abstracts or executive summaries of the remaining 74 publications were searched by one author (JM) to classify the characteristics of the publications, with data entered into an Excel spread sheet.

### Classification of studies

The tools were categorised according to their general characteristics: 1) Indigenous-specific or not; 2) tool type; 3) Ottawa Charter strategy focus (building healthy public policy, creating supportive environments, strengthening community actions, developing personal skills, and reorienting health services [13]); 4) Indigenous health promotion principles articulated (using the words of study authors but including cultural competence, community engagement and ownership, partnerships, holism, best practice, capacity development, sustainability, leadership, consultation and participation) 5) health issue; and 6) year of publication (calculated by the date of publication or date posted on the website). Where many diverse concepts and terms were used, similar terms and concepts were clustered.

### Assessing the implementation of health promotion tools

Successful implementation of a health promotion tool or program requires three elements as characterised in the Promoting Action on Research Implementation in Health Services (PARiHS) framework: evidence of its potential effectiveness; consideration of the organisational, community and broader context in which it is to be implemented; and the methods by which its delivery will be facilitated [14]. PARiHS was considered by Brands et al. [15] as one of the most accessible and flexible frameworks for Indigenous health implementation in their review of the applicability of health implementation literature in Indigenous settings because it was simple yet encompassed evidence, context and facilitation. The three elements of the PARiHS framework were used to categorise the implementation of the Indigenous health promotion tools documented in the 74 publications.

Evidence was considered to be the form of knowledge that was implemented through tools. Evidence was derived from research, clinical expertise, and/or local and Indigenous knowledge from clients [16,17]. The evidence of tool implementation was characterised according to: 1) type of evidence used to inform tool development; and 2) whether the tool was evaluated or described (that is, whether there was evidence of its potential effectiveness). Context was defined as the environment or setting in which the proposed tool was to be implemented [18]. To determine the contexts within which tools were implemented (or intended to be implemented), the tools were categorised by the: 1) broad policy and community context; 2) organisational context; and 3) individuals involved in implementing the tool. Facilitation was defined as the process by which change managers helped others towards achieving particular goals, encouraged others, and promoted action to help people change their attitudes, habits, skills, ways of thinking, and working [18]. To determine the methods of facilitation used, the tools were characterised by the: 1) implementation process; 2) facilitators and barriers to implementation; and 3) recommendations for improving uptake and implementation.

### Assessing the effects of health promotion tools

To determine the effectiveness of tools for Indigenous health promotion improvement, impact evaluation studies were categorised by: 1) effects; 2) study quality; and 3) publication type. The methodological quality of quantitative studies was assessed using the Dictionary for Effective Public Health Practice Project (EPHPP) tool [19]. Sections A to F (A. internal selection bias; B. study design; C. confounders; D. blinding; E. data collection methods; and F. withdrawal and drop-outs) were coded weak, moderate or strong, consistent with the component rating scale of the Dictionary. For Sections G (intervention integrity) and H (analyses) descriptive information was recorded, in line with the Dictionary recommendations. The quality assessment of qualitative studies was assessed using the Critical Appraisal Skills Program (CASP) tool [20]. To assess the study quality of those using mixed-methods design, the qualitative and quantitative components were assessed separately using the aforementioned tools. Assessments of quality were made by two authors (RB and JM) with inter-rater reliability assessed as 94%. The peer review of publications was considered a second quality measure given a correlation in public health between peer review and assessments of research influence [12].

## Results and discussion

### Characterising health promotion tools

**Figure 2:**
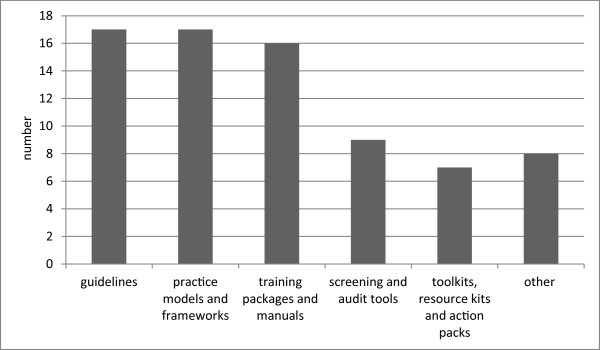
Summary of types of health promotion tools identified in the literature.

The review found 74 publications that described or evaluated tools designed to enhance Indigenous health promotion practice. They included 60/74 (81%) Indigenous Australian-specific tools; five non-Indigenous specific tools that were tailored for Indigenous Australians, nine tools that were non-Indigenous specific and not tailored, and one Canadian Indigenous tool. Figure 2 outlines the number of guidelines, practice models or frameworks, training packages/manuals, toolkit/resource kit/action packs, screening tools, audit tools, handbooks, measurement tools, checklists and networks documented. These tools were used as part of health promotion programs and services; the programs and services themselves were not considered tools.

**Figure 3:**
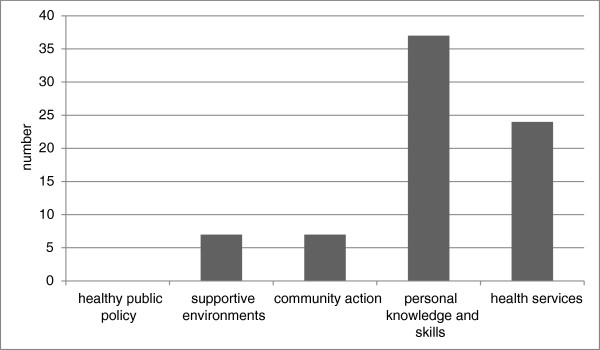
Health promotion strategies targeted.

The focus of tools was also diverse with regard to the five health promotion strategies outlined in the Ottawa Charter (Figure 3). However, few of the tools targeted the upstream approaches implicit in the holistic view of Indigenous health. For example, there were no documents that provided tools on the processes associated with policy advocacy, only seven documents provided tools for creating settings and supportive environments and a further seven for improving work on community action/ development. In contrast, 37 (49%) were oriented towards improving the downstream factors through health information/education for personal skills development and 25 (34%) tools were associated with reorienting health services and disease prevention (Table 1). This suggests that there has been relatively less emphasis on tools implementation to support the broader policy, environmental or community-based and holistic Indigenous health approaches than those targeting more individualised health service approaches.

**Table 1:** Contexts and settings in which identified health promotion tools are intended to be implemented

**Geographic locations**	**Organisational settings**	**Individuals**
National 45 (61%)	Primary health care* 31 (42%)	PHC workforce** 32 (42%)
State or Territory 20 (27%)	Community organisations 17 (23%)	Community members12 (16%)
Regional or local 9 (12%)	Health promotion 14 (19%)	HP Officers*** 11 (15%)
	Universities 4 (5%)	Policy makers 5 (7%)
	Training organisations 1	Community/welfare workers 5 (7%)
	AOD service 2	AOD, tobacco workers*** 4 (5%)
	Mental health service 1	Mental health workers*** 1 (1%)
	General 8	Sexual health workers*** 1 (1%)
		Indigenous academics 1 (1%)

*Includes ACCHOs, General Practice and Medicare Locals.

**Includes medical practitioners, nurses and AHWs.

***Sometimes part of the PHC workforce.

Almost all publications documenting Indigenous-Australian-specific tools (57/60 or 95%) incorporated some description of Indigenous principles or ethical guidelines which informed their development or implementation. Most commonly reported were the principles of cultural competence, community engagement and empowerment, partnerships, holism, best practice, capacity development, and sustainability. Less commonly cited were the principles of incremental change; communication; community leadership; social and kinship relations; safety; harmony with country, equity, lawfulness, use of pictures, use of Aboriginal language, simplicity, prompt response, consensus, enhancing current investments, and fun/passion/sharing.

**Figure 4:**
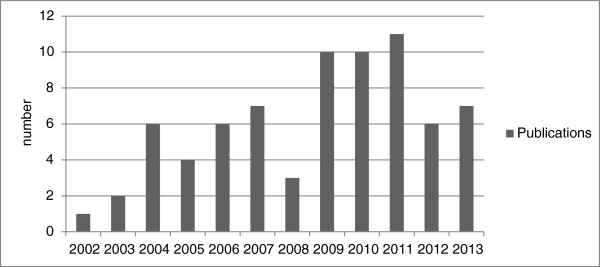
Date of publications.

The three most common health issues which together accounted for 38% of the tools, were mental health and alcohol and drug issues (11/74 or 15%); healthy lifestyle/chronic disease prevention (11/74 or 15%); and nutrition (6/74 or 8%). But the health issues were more notable for their diversity than similarity. In addition was documentation of tools to strengthen Indigenous environmental health/drinking water; physical activity; tobacco control; maternal and child health; women’s health; social and emotional wellbeing; men’s health, child injury, sexual health, ear health, musculoskeletal health, remote health and dementia/cognitive impairment.As shown in Figure 4, more than half of the publications were published in the last five years (44/74 or 59%).

### Assessing the implementation of health promotion tools using the PARiHS framework

The three elements of the PARiHS framework were examined for each health promotion tool. These three elements (evidence, context and facilitation) are considered in turn.

#### ***Evidence***

Hearteningly, all health promotion tools were developed, adapted, or updated in reference to evidence. The type of evidence consulted varied from research and clinical expertise to community preference. While it is critical that Indigenous knowledge was incorporated into the design of tools, only 52/74 (70%) publications specified that community members were consulted or collaboratively involved in developing or adapting tools. Other commonly mentioned sources of evidence for tool development were reviews of the literature (15/74 or 20%) or consultation with health practitioners (12/74 or 16%).

Evidence was important in the development and implementation of tools because it improved users’ confidence in the utility of the tool and their willingness to apply it to improve health. The value of evidence was illustrated by Davidson et al.’s [21] evaluation of a collaborative model of cardiovascular education for Indigenous Health Workers. Confidence in the thorough process of combining research, clinical expertise, and local and Indigenous knowledge in the development and delivery of the course partnership model led to significant increases in Indigenous Health Workers’ knowledge and assurance for collaboration, skill development, cultural competence and access to mentorship and expertise. The knowledge incorporated within the course curriculum was consistent with national competency standards and based on a prior nationally accredited training course in cardiovascular health for Indigenous Health Workers, but tailored and delivered with contributions from the local partners, including Indigenous presenters. As well, the development of partnerships led to increased knowledge of Indigenous health in the mainstream health settings [21]. Similarly, Whiteside, Tsey, Crouch, & Fagan [22] found that a facilitated community participation strategy which integrated evidence from sexual health experts and local people in two North Queensland communities produced signs of a changing discourse around sexual health.

Further, the impacts of tool implementation were evaluated in only 11/74 (15%) studies. In the remaining 63/74 (85%) publications, the studies described or evaluated the process of implementation of tools. Process evaluations found that screening and measurement tools were validated, reliable and culturally appropriate; guides were updated, supported by staff and/or implemented; and training packages were feasible, relevant, culturally acceptable, likely to be cost effective and/or accredited. However, the dearth of impact evaluation studies means that there is little evidence for whether tools work to improve Indigenous health promotion.

#### ***Context***

Overall, the reviewed studies identified a huge diversity of geographical and organisational contexts, and individual practitioners involved in Indigenous health promotion (Table 1). The importance of attending to context when implementing health promotion tools was exemplified by D'Espaignet, Measey, Carnegie, and Mackerras [23]. They concluded that there was a need to better understand how the Strong Babies, Strong Culture Program differed across two groups of Northern Territory communities to reduce birth weights in one group, but not in the second group. Due to the lack of descriptors in the primary studies, however, it was not possible to determine which contextual factors influenced effective implementation or how.

As shown in Table 1, only, 9/74 (12%) of tools were developed and tailored for regional or local implementation in health service and/or community settings, whilst the majority of tools were designed for use nationally or for State or Territory-wide use. Within these geographical settings, there was little acknowledgement in studies of the specific influence of broad macro political, technological and ideological settings on tool implementation.

The most common intended organisational settings for the use of tools were primary health care and community-based organisations. As well, tools were designed for specific use in health promotion projects, by policy makers, within research projects, within the alcohol and other drug sector, in the mental health sector and for use by a training organisation. The diversity of organisational contexts within which Indigenous health promotion tools were implemented is both a strength and challenge within health promotion. The holistic Aboriginal view of health promotion requires coordinated, inter-sectoral partnerships – with leadership from Aboriginal community controlled health bodies as well as government and non-government sectors [5]. However, by making health promotion everyone’s business, there is a risk that it becomes nobody’s business. There was little or no evidence of systematic development of tools targeting different groups around a particular health promotion focus, for example, a suite of tools or resources about sugary drinks that targeted consumers, primary health care practitioners, store managers, community councils and policy makers. Different audiences were targeted, but that they were targeted in an ad hoc rather than systematic way.

Consistent with the diverse organisational contexts, individual tool implementers were also diverse. They included primary health care workers, community members; health promotion officers; policy makers, board members and managers; community/welfare workers; specialist health practitioners; and Indigenous academics.

#### ***Facilitation***

**Figure 5:**
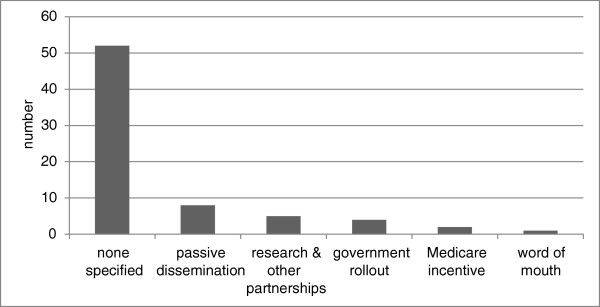
Strategies for facilitating implementation of health promotion tools.

The importance of facilitation was illustrated by Hunter, Brown and McCulloch [24] who found that the distribution of a clinical guideline alone was not sufficient to ensure use. The use of the guidelines, particularly by medical practitioners, was increased when expert clinicians and workers in the area of Indigenous primary care and substance use introduced them through a series of workshops. The introduction and dissemination of the guidelines by acknowledged (Indigenous and non-Indigenous) experts enforced credibility, provided opportunity for facilitated discussion of issues, and resulted in improved acceptance of the role of health practitioners in dealing with alcohol problems. Other studies also noted the importance of facilitated implementation rather than passive dissemination methods, including the importance of Indigenous leadership and involvement of Indigenous people. For example, Tsey et al. [25] found that a participatory action research (PAR) process implemented with the Yarrabah men’s group reinforced the modest but significant change in the men’s personal development and growth and in their response to family responsibilities. Nevertheless, other studies cited considerable variability of implementation across sites. For example, the Centre for Appropriate Technology [26] found that the adoption of the tools for improving water quality remained patchy and unstructured despite their free access, and that continued promotion and investment were required to maintain momentum.Nevertheless, the review showed a lack of attention to how tool implementation was facilitated with only 22/74 (30%) publications specifying facilitation (or intended facilitation) methods. For the studies which did describe facilitation methods, the most common strategy was passive dissemination (their free availability through websites). Additionally, the implementation of tools was facilitated through research or other partnerships, government-funded rollouts, Medicare incentives or requirements, and word of mouth (Figure 5). For at least 70% of tools, whether and how they were being utilised was not specified; neither was there information about how health promotion practitioners learnt about their availability and/or how methods of tool dissemination could be improved.

Eighteen of the 74 (24%) studies described barriers to implementation including the lack of public availability of the tool; effort, motivation and capacity of workforce required to apply the tool, and lack of funding. Implementation was facilitated by the simplicity, attractiveness, relevance and availability of the tools themselves, and also by partnerships and networks, recommendations by Indigenous and health leaders, health practitioner confidence, skills and knowledge; supportive environments; pilot programs; and requirements or incentives. Recommendations or guidance relevant to implementation were provided in 26/74 (35%) publications as predominantly general statements such as a need to explore ways of making tools available to other health professionals.

### Assessing the effects of health promotion tools

The impacts of tools were evaluated in only 11/74 (15%) publications. Table 2 provides the details of the eleven impact evaluation studies in terms of the tool type, their implementation (evidence, context and facilitation), their effects and the evaluation design and quality. Of the small sample of evaluated studies, training packages (4) and guidelines (3) were most commonly the tools that produced health effects. Additionally measurement tools, practice frameworks and screening tools produced effects. However, the tools were developed in reference to different types of evidence, implemented in assorted contexts and facilitated in various ways. That is, the review did not identify a standard recipe for effectively implementing Indigenous health promotion tools.

**Table 2:** The evidence, context, facilitation and impacts of evaluated health promotion tools, and quality of evaluations

**Program**	**Type of tool**	**Evidence for tool development**	**Context specified**	**Facilitation strategies**	**Impacts or outcomes**	**Evaluation design and quality**
Strong Women, Strong Babies, Strong Culture [23]	Practice framework	Research literature; professional practice; pilot program	Community-based - program delivery in Northern Territory	Employment of senior Aboriginal women; other strategies not specified	Increased birth weights in one group, no significant change in the second group	Quantitative Strong
Healthy weight program/ Living strong program [27]	Health screening tool and training package	Reviewed the literature	QLD	Delivered by Indigenous health workers, community health staff and non-government health staff	Majority of participants achieved reductions in body weight and waist/hip circumferences; and modest positive change in lifestyle behaviours	Qualitative Weak
Coping skills for partners of alcoholics [28,29]	Training package	Based on U.S. program	Alcohol rehabilitation settings	Identified as effective but there was no documentation of actual implementation	Lower depression levels, partner drinking and relationship violence	Quantitative Strong
		Research literature				
		Professional practice				
Participatory community planning [30]	Guidelines	Reviewed the literature	Remote community of Mapoon	Participatory planning	Influenced town plan, and likely health benefits	Qualitative Strong
Community water planner field guide project [26]	Guidelines	N/A	National	Distributed	Improved management for small water supplies	Qualitative Weak
Integrated Yarn model for sexual health training [22]	Practice framework	Knowledge from Indigenous sexual health workers and other health professionals	North QLD	Training	Useful framework for guiding practice	Qualitative Moderate
Yarrabah men’s group tool for measuring improvements in men’s behaviour [25]	Measurement tool	Research partnership with a community –controlled health organisation	Rural community of Yarrabah	Through research partnership	Men made small improvements towards their stated goals	Qualitative Moderate
Measurement tool for workforce-rated improvements in organisational change [31]	Measurement tool	Research partnership with a community –controlled health organisation	Cairns and Cape York	Through research partnership	Monitoring of organisational change, improved staff wellbeing and empowerment	Qualitative Moderate
Clinical management of alcohol-related problems [24]	Guidelines	National recommendations	National	Distribution through standardised workshops for general practitioners, and opportunistic provision and on request	Appropriate introduction increased use and positively influenced willingness to engage	Mixed
						Strong (qual)
						Moderate (quant)
Cardiovascular education program [21]	Training package	Steering Committee chaired by AHWs	NSW	Partnership model for collaboration	Knowledge and confidence scores increased and students placed a very high value on clinical visits	Mixed
						Strong (qual)
						Weak (quant)
Smoking cessation training program [32]	Training package	Research literature, piloted in north Queensland	NSW	Implemented by government.	Built self-reported knowledge and skills and confidence in brief intervention	Quantitative Moderate

The studies provide diverse examples of tool implementation, suggesting that facilitating evidence-informed tools is feasible in community organisations and groups, primary health care services, training organisations and specialist alcohol rehabilitation and other services. The delivery of tools by Indigenous community members, Indigenous health workers and other health professionals, expert mental health and alcohol and other drug advisors, government trainers, and research partnerships suggested that facilitation by diverse individuals was also feasible. Facilitated processes were more commonly effective than passive dissemination methods.

The documented health, environmental, community, organisational and health care improvements from the eleven impact evaluation studies were promising, suggesting that the implementation of tools can be an effective strategy for strengthening evidence-informed Indigenous health promotion practice. The health impacts of tool implementation comprised improvements in birth weight, reductions in body weight and waist/hip circumferences and lower depression levels, partner drinking and relationship violence. For example, D’Espaignet et al. [23] found significant improvements in birth weight following the introduction of the Strong Women, Strong Babies, Strong Culture Program in one group of Aboriginal communities in the Northern Territory, although there was no significant change in the second group. Fredericks et al. [27] found that the majority of participants in a healthy weight/living strong program achieved reductions in body weight and waist/hip circumferences; and also showed some modest positive change in terms of their lifestyle behaviours. Although not evaluated in Indigenous Australian settings, the coping skills training evaluated in the U.S. study by Rychtarik and McGillicuddy [28] was identified as being relevant to Indigenous Australians [29]. The study found that coping skills training for women who were distressed by their partners’ untreated alcoholism resulted in lower depression levels than delayed treatment and that effects were maintained at 12 months. Partner drinking and relationship violence also decreased from pretreatment to follow-up.

Consistent with the health promotion strategy of creating supportive environments, improvements in physical infrastructure and housing, and water management were found. Moran’s [30] qualitative study of participatory planning in the north Queensland community of Mapoon found that the plan improved physical infrastructure and housing, but had mixed success in terms of community development. Similarly, the Centre for Appropriate Technology [26] found improved management for small water supplies.

The use of tools also impacted community and organisational processes such as a changing discourse around sexual health, change in men’s personal development and growth and in their response to family responsibilities, and shifts in organisational culture and group cohesion. Whiteside et al. [22] established that a community participation strategy in two North Queensland communities produced signs of a changing discourse around sexual health. Tsey et al. [25] demonstrated that a participatory action research (PAR) process implemented with the Yarrabah men’s group reinforced the modest but significant change in the men’s personal development and growth and in their response to family responsibilities. McEwan et al. [31] also found that participatory action research and empowerment strategies used in a change management process with the Apunipima Cape York Health Council facilitated shifts in work culture and group cohesion towards achieving the community controlled health organisation’s vision of being an effective lead agency for Indigenous health reform in Cape York.

Finally, health care impacts as a result of tool implementation included a willingness by primary health practitioners to engage with alcohol-related problems; collaboration, skill development, cultural competence and access to mentorship and expertise; and increased knowledge, skills and confidence to implement smoking brief intervention. The distribution of a clinical guideline (the National Recommendations for the Clinical Management of Alcohol-Related Problems in Indigenous Primary Care Settings) positively influenced willingness to engage with alcohol-related problems as part of primary clinical care [24]. Davidson et al. [21] demonstrated that a partnership model between key education providers, policy makers, non-government organisations, the local area health service and Aboriginal community controlled organisations significantly increased Aboriginal Health Workers’ knowledge and confidence for collaboration, skill development, cultural competence and access to mentorship and expertise. And Hearn et al. [32] found that a culturally specific smoking cessation training program for health professionals increased professionals’ knowledge, skills and confidence to provide an evidence-based quit smoking brief intervention to Aboriginal clients, but no changes were reported in smoking or intention to quit.

The quality of evaluation studies, measured using EPHPP (for quantitative studies) and CASP (for qualitative studies) quality assessment tools, however, was strong for only five of the studies (attached as Additional file 3: Table S1). Further, while 9/11 impact evaluation studies had been published in peer-reviewed publications, only 29/74 (39%) of all reviewed studies had been described or evaluated in peer-reviewed publications. Overall, the small number and a generally moderate quality of evaluation studies means that little is known about the effects of using health promotion tools. This suggests the importance of long-term investment in research studies to review the current use of health promotion tools and the factors that are likely to enhance their implementation.

### Potential limitations

The definition of a health promotion tool was variously defined within publications and relevant studies may have been missed. We therefore applied a clear pre-determined definition within our systematic search for tools. The high level of agreement between blinded coders and the process of negotiated consensus to deal with discrepancies helped to confirm studies included. Potentially useful information about context, individuals and implementation processes of tools may have been described in studies that were excluded.

The use of authors’ knowledge of health promotion tools to identify additional tools was justified since all authors were Indigenous Australian health promotion researchers with broad knowledge of the field, and two are Aboriginal. However this strategy may have led to inclusion of more of the tools with which authors were locally familiar, and hence an oversampling of tools from the author’s locations in the Northern Territory, Queensland and Victoria. Given that only 8/74 publications were identified through authors’ knowledge, this potential bias was small.

## Conclusions

The review found that Indigenous health promotion tools, including tools developed specifically for the Indigenous Australian population, were widely available. Tools were all developed in reference to some evidence; most commonly through consultation with community members and/or health practitioners or reviews of the literature. Tools were implemented through facilitated processes and passive dissemination methods by Indigenous community members, Indigenous health workers and other health professionals, expert mental health and alcohol and other drug advisors, government trainers, and research partnerships. The organisational settings for tool implementation were also diverse, including community organisations and groups, primary health care services, training organisations and specialist alcohol rehabilitation and other services. However, the documentation of how tools were intended to be or were implemented was poor, being reported in only 30% publications, and only 15% publications evaluated the impacts of using the tools. Evaluations found that the implementation of tools resulted in health, environmental, community, organisational and health care improvements, but the quality of impact evaluation studies was strong for only seven percent of studies.

Health promotion has been critiqued internationally as only “a pale version of what it could be” [33]. Contributing to the gap between health promotion potential and the widespread implementation of effective health promotion practice are the deficits in our knowledge about whether available tools are effective and how they were implemented in practice. The small number and generally moderate quality of implementation and evaluation studies means that little is known about how tools work to strengthen Indigenous health promotion, how or where health promotion tools are being utilised, and the effects of use and how implementation could be improved. The dominance of descriptive studies and poor quality of evaluations found in this review is consistent with that of other reviews of the “sorry state” of Indigenous health evidence, especially in health promotion [29,34-39]. There is a need to examine how research can better contribute to sustainable health promotion outcomes for Indigenous communities.

To justify further investment in health promotion efforts, policy makers and practitioners are pressured to demonstrate benefits, particularly in terms of reduced costs, improved value for money or improved health outcomes [40]. This review suggests that in addition to evaluating impacts, evidence, context and facilitation are important to the effective implementation of Indigenous health promotion tools. The PARiHs framework offers a useful tool for use by health promotion teams or policy makers to assess and prioritise the factors which affect implementation. The findings of this review suggest that rather than continuing to investing in tool development [41], practitioners, policy makers and researchers could instead focus attention on strengthening health promotion tools by evaluating and publishing the results. Evaluation should include the influence of factors such as changes in workforce structures, policy directions, and funding on the implementation and effects of existing tools. There is a need for long-term investment in research studies to review the current use of health promotion tools and the factors that are likely to enhance their implementation.

## Competing interests

The author(s) declare that they have no competing interests.

## Authors’ contributions

JM and KT made substantial contributions to the conception and design, JM acquired the data and JM and RB analysed and interpreted data. All authors critically revised drafts of the manuscript and approved the final manuscript.

## Pre-publication history

The pre-publication history for this paper can be accessed here:

http://www.biomedcentral.com/1471-2458/14/712/prepub

## Supplementary Material

Additional file 1 Protocol for Lowitja tools review. Click here for file

Additional file 2 PRISMA Checklist. Click here for file

Additional file 3 Spreadsheet. Click here for file
