# Supplementary material for: The characteristics, implementation and effects of Aboriginal and Torres Strait Islander health promotion tools: a systematic literature search
Source: BMC Public Health. 2014 Jul 11;14:712. doi: 10.1186/1471-2458-14-712 (PMC4227054; doi:10.1186/1471-2458-14-712)
Supplement: Additional file 1 — Protocol for Lowitja tools review. [file 1471-2458-14-712-S1.docx]

# Protocol for systematic search: What are Aboriginal and/or Torres Strait Islander health promotion tools and what evidence is there that they work?

**1.** **Background**

In September 2011, The Lowitja Institute Healthy Communities and Settings Program called for expressions of interest for a Health Promotion Capacity Building project. Stage one required a review of the literature and programs to scope current Aboriginal and Torres Strait Islander health promotion tools and possible gaps and opportunities for the development of further tools. This formed the basis for stages 2–4 which focused on enhancing Aboriginal and Torres Strait Islander health promotion capacity by adapting, developing, piloting and evaluating available tools, resources or training in order to produce a suite of tested health promotion tools, resources and/or training for use by health promotion officers.

Stage one was completed by ([Wise, Angus, Harris, & Parker, 2012](#_ENREF_16)). The intent of their scoping study was to review Indigenous health promotion tools. They defined health promotion tools broadly as: ‘defined techniques, guidelines and implementation processes designed to achieve a particular purpose’ (p.6). The purpose of health promotion was described as planned positive social change in groups, communities and societies in order to promote, protect, and sustain good health and wellbeing. A total of 93 tools were identified in the scoping study. These tools included broad policy frameworks and guidelines, sources of data, information sources for broad background principles, descriptive reports of funded projects for specific health issues, evidence, and learnings to inform Aboriginal and Torres Strait Islander health promotion programs. The “tools” did not necessarily directly guide pragmatic efforts to plan, implement or evaluate health promotion initiatives in local health care services or other settings. Wise et al. (2012) did not attempt to evaluate the quality of evidence underpinning the identified health promotion tools.

**2. Rationale**

In attempting to implement health promotion, Indigenous primary healthcare and other services are faced with poor literacy, limited health promotion training, cross-cultural difference, social disadvantage and complexity, comorbidity and high rates of chronic disease. There is little evidence of tools or strategies which could assist Indigenous health services and other organisations to make informed decisions regarding which health promotion strategies they should implement or best to integrate effective strategies into routine use ([Clelland, Gould, & Parker, 2007](#_ENREF_3); [Clifford, Doran, & Tsey, In press](#_ENREF_4); [Sanson-Fisher, Campbell, Perkins, Blunden, & Davis, 2006](#_ENREF_15)). Systematic reviews have consistently found little intervention research, which is necessary for designing, implementing and evaluating the costs and benefits of Indigenous-specific health strategies ([Clelland, et al., 2007](#_ENREF_3); [Clifford, et al., In press](#_ENREF_4); [Garvey, 2008](#_ENREF_5)), and little Indigenous implementation research ([J. McCalman et al., 2012](#_ENREF_8)). Scholars have also argued that there is a need for distinct Indigenous Australian perspectives on the nature of the research evidence, and recognition of distinct concepts of Indigenous health and wellbeing ([Gooda, 2010](#_ENREF_6); [Prout, 2012](#_ENREF_13)). While access to health promotion tools has increased considerably with use of the internet, the quality of these tools varies considerably ([Atack & Luke, 2012](#_ENREF_1)). The poor state of the evidence and complexity of cross-cultural understandings has hampered the efficient use of existing health promotion tools and increased the likelihood that delivery of ineffective health promotion will persist. Paul, Sanson-Fisher et al. (2010) described the “sorry state” of the Indigenous health evidence base and cautioned that before commencing on major research efforts in an important area such as Indigenous health promotion, attention needs to be paid to analysing the existing knowledge output in order to identify the evidence gaps.

**3. Method.**

The James Cook University (JCU) team proposes to contribute to a tri-university collaboration for stages 2-4 of the Health Promotion Capacity Building project by using a three-part method to answer three research questions:

1) what are the characteristics of tools designed to promote Indigenous Australian health;

2) how and where have tools been developed and implemented; and

3) what were the effects of tool implementation on Indigenous health promotion improvement?

First, building on Wise et al.’s scoping study, we will construct a short-list of evidence-informed Indigenous health promotion tools. We will draw from the Wise et al. (2012) review as well as the reference lists of 19 other existing reviews of Indigenous health promotion evidence ([see J. McCalman, et al., 2012](#_ENREF_8)), and an existing database of 1393 Indigenous health programs and services ([J McCalman et al., 2012](#_ENREF_7)) to quantify and describe the nature of the evidence for Aboriginal and Torres Strait Islander health promotion tools. The review will determine how many health promotion tools are available for use in Aboriginal & Torres Strait Islander communities and settings, what tools are available across the spectrum of health promotion, what health issues are they focussed on, what we know about how they have been used, and the evidence for their quality and effectiveness (including cost effective or value for money). We define health broadly according to the Indigenous Australian definition which includes physical, mental, emotional and spiritual wellbeing ([National Aboriginal Health Strategy Working Party (NAHS, 1989](#_ENREF_10)). Health promotion is defined as “the process of enabling people to increase control over the determinants of health and thereby improve their health” ([Nutbeam, 1986](#_ENREF_11)). We narrow Wise’s definition of tools as: structured step-by-step guides, instruments, packages, frameworks or resources which are designed for applying the knowledge and skills needed to plan, implement or evaluate an Aboriginal and Torres Strait Islander health program or improve an existing one ([Otoo, Agapitova, & Behrens, 2009](#_ENREF_12)). Examples include guidelines, training manuals, program manuals, resource manuals, practice frameworks, checklists, community action packs, audit tools, toolkits, standards and programs. The findings of the review will be workshopped with the cross-university project team in relation to our three programs of work. Gaps will be identified and ameliorated.

Second, we will organise forums with Indigenous health promotion practitioners to explore what the evidence means for health promotion practice. Indigenous health promotion practitioners are defined as health professionals who specialise in maintaining and improving the health of Aboriginal and Torres Strait Islander peoples and reducing the health inequities experienced by Aboriginal and Torres Strait Islander peoples through the action areas articulated in the Ottawa Charter: building healthy public policy, creating supportive environments, strengthening community action, developing person skills, and reorienting health services ([Australian Health Promotion Association, 2009](#_ENREF_2)). Tools can be used to support the roles of Indigenous health promotion officers, such as planning, development, implementation and evaluation of health promotion policies and projects using strategies such as health education, mass media, community engagement and community development, advocacy and lobbying, social marketing, health policy, structural and environmental strategies and workforce development and capacity strengthening ([Australian Health Promotion Association, 2009](#_ENREF_2)). Participant groups will be recruited to the forum in conjunction with extant research projects and the broader cross-university project team and are likely to include: 1) community-based Indigenous health promotion practitioners from north Queensland research partnerships (in Cairns); 2) health promotion practitioners associated with an allied health capacity enhancement project and the University of Melbourne/Shepparton project (in Melbourne); and 3) health promotion practitioners from Northern Territory health services (in Darwin).

Indigenous health promotion practitioners will be asked to identify which of the identified tools they have used, what has worked in their practices, what other tools they have found useful, the relative importance of and nature of the evidence used when selecting tools, the relevance, feasibility and practicality of uptake and implementation of identified evidence-informed strategies for Indigenous health settings, and to identify opportunities and options for the adaptation, development, further piloting and evaluation of available tools. The forums will be documented and data thematically analysed to determine the enablers, barriers and benefits of uptake and implementation of health promotion tools. Thus, we will document a suite of tested health promotion tools used by Indigenous health promotion officers, and identify the gaps and needs for the further development of accessible packages of feasible innovations.

Third, we will continue to build the evidence base for extant health promotion tools to enhance sustained Indigenous health promotion workforce capacity. A select number of evidence-informed health promotion tools will be packaged and made available online, such as through the Indigenous Health InfoNet and Lowitja website. The selection of these packages will be informed by the forums. The feasibility of methods for tracking usage of the online resource will be explored, including counts of online downloads or online survey data.

**3. Data and analysis**

First, the review builds on the scoping study (Wise et al. 2012) augmented by hand-searching the reference lists of 19 reviews of Indigenous Australian health and wellbeing interventions and a database of 1393 Indigenous health programs and services developed for a previous systematic review of the transfer of Indigenous Australian health services and programs ([J McCalman, et al., 2012](#_ENREF_7)). The time period for the review is 2002-2012 – this ten year period provides comprehensive coverage of relevant initiatives. The data will be extracted from studies using a customised on-line data extraction form. The characteristics of publications will be categorised by: 1) tool type 2) 1st author & year; 3) publication type; 4) study design/evidence informed?; 5) location; 6) health issue; 7) health promotion technique; 8) target population; 9) outcomes or effects found; 10) study quality (intervention studies only). The data extraction will be undertaken by one researcher and categorisation of 10% studies validated by a second researcher, particularly for any data that requires numerical calculations, or is subjective.

Methodological quality for intervention studies will be assessed using the Dictionary for the Effective Public Health Practice Project Quality Assessment Tool for Quantitative Studies ([McMaster University, 2008](#_ENREF_9)). Sections A to F (A. selection bias; B. study design; C. confounders; D. blinding; E. data collection methods; and F. withdrawal and drop-outs) will be coded weak, moderate or strong, consistent with the component rating scale of the Dictionary. For Sections G (intervention integrity) and H (analyses) descriptive information will be recorded, in line with the Dictionary recommendations. A selection of 10% of intervention studies will be re-evaluated using the Dictionary checklist by another researcher blinded to the results of the initial ratings, to cross-check the ratings performed by the first researcher. Again, differences between data extractors will be resolved by negotiation to consensus. The findings of the reviews will be workshopped with the cross-university project team in relation to our three programs of work, with gaps identified and ameliorated.

Second, the views of workshop participants will be documented and summarised. Key findings of the workshops will be synthesised with those of the literature review using Framework Analysis, a highly structured approach to organising and analysing data (e.g. indexing using numerical codes, rearranging data into charts) from different sources e.g. primary and secondary data ([Ritchie & Spencer, 1994](#_ENREF_14)). The main outcome will be a matrix of evidence-informed strategies for improving the implementation of health promotion innovations in Indigenous health services, provisionally rated according to their relevance and suitability from a service and practitioner view point. This will enable the identification of high priority health promotion tools.

Third, the priority health promotion tools will be packaged and made available online, such as through the Indigenous Health InfoNet and Lowitja website. Contributions will include the collaborative development of an e-book that showcases best practice models and approaches in Indigenous health promotion. Provisionally titled “ Beyond the flipchart: best practice approaches in Aboriginal and Torres Strait Islander health promotion today”, the e-book will likely be housed on the Australian Indigenous Health InfoNet and will consolidate what we have learnt about strengthening health promotion across the three research teams, as well as providing the three case studies of the research-practice/policy implementation journeys from the HPCQI, FWB and community engagement/action research processes that have been developed in collaboration with relevant Aboriginal service staff. A simple template will be provided to enable other Indigenous health promotion practitioners to incrementally add further stories of health promotion endeavours. The content will outline the processes by which workforce and organisational capacity has been strengthened and service efficiency and effectiveness have been improved through partnerships between Indigenous community organisations, other health promotion stakeholders, and researchers. The feasibility of methods for tracking usage of the online resource will be explored, including counts of online downloads or online survey data. As well, a number of publications will be produced for peer-reviewed journals related to how Indigenous health promotion workforce capacity enhancement.

**8. Ethical issues**

Because the data used will be secondary data in the public domain, we do not envisage ethical issues. The data from workshops will be subject to normal ethical processes.

**9. Limitations**

Like Wise et al (2012), we exclude resources (information about health promotion strategies, knowledge, principles and experiences) and training (accredited and non-accredited training courses and sessions designed to increase knowledge and skills). We also exclude broader guides such as policy frameworks. We consider that tools are likely to provide direct guidance for health promotion practice, and that the focus of our efforts in stages 2-4 should be on exploring how best to use them to enhance health promotion capacity.

**10. Deliverables**

Two key deliverables will emanate directly from this research.

First, two papers will be produced for leading peer-review journals. The first will report on the analysis of the health promotion tools literature. The second will report on the findings of the forums regarding the role of evidence in supporting and sustaining health promotion capacity. The focus will be the match between the scientific evidence and the feedback from Indigenous health promotion practitioners. The target audience for the papers will be Indigenous health promotion practitioners and policy makers.

Second is the collaborative development of an e-book that showcases best practice models and approaches in Indigenous health promotion (described above).

**10. Milestones**

The time estimates and milestones for the major steps of the review are outlined below.

| Due date | Milestone | Expected output/outcome as designated in project proposal | Amended output |
| --- | --- | --- | --- |
| March 31, 2014 | Protocol confirmed. | Collaborative paper submitted to peer-reviewed journal  E-book authors identified; content confirmed. Negotiations with HealthInfonet | Discussion paper - The levels of evidence for health promotion tools for Aboriginal and Torres Strait Islander people |
| August 31 2014 | Mid-term think tank (2)  Draft e-book | Reflection on key themes relating to health promotion practice across the four groups sites and implications for broader health promotion practice | 2 x draft papers for peer reviewed journal completed  First draft of e book “chapters”; template for online contribution designed. |
| June 30 2014 | Showcase event e-book launched | e-book available online | e-book available online |

**References**

Atack, L., & Luke, R. (2012). The impact of validated, online health education resources on patient and community members' satisfaction and health behaviour. *Health Education Journal, 71*(2), 211-218.

Australian Health Promotion Association. (2009). Core competencies for health promotion practitioners. Maroochydore, QLD: University of the Sunshine Coast.

Clelland, N., Gould, T., & Parker, E. (2007). Searching for evidence: what works in Indigenous mental health promotion? *Health Promotion Journal of Australia, 18*(3), 208.

Clifford, A., Doran, C., & Tsey, K. (In press). A critical review of suicide prevention interventions targeting Indigenous peoples in Australia, new Zealand, United States and Canada. *BMC Public Health*.

Garvey, D. (2008). A review of the social and emotional wellbeing of Indigenous Australian peoples – considerations, challenges and opportunities Perth: Australian Indigenous Health InfoNet

Gooda, M. (2010). *Social justice and wellbeing*. Paper presented at the AIATSIS Seminar Series: Indigenous wellbeing, Canberra.

McCalman, J., Tsey, K., Clifford, A., Earles, W., Shakeshaft, A., & Bainbridge, R. (2012). Applying what works: a systematic review of the transfer of promising services and programs *BMC - Public Health, 12*(600). doi: 10.1186/1471-2458-12-600

McCalman, J., Tsey, K., Clifford, A., Earles, W., Shakeshaft, A., & Bainbridge, R. (2012). Applying what works: A systematic search of the transfer and implementation of promising Indigenous Australian health services and programs. *BMC Public Health, 12*(600).

McMaster University. (2008). Effective Public Health Practice Quality Assessment Tool for Quantitative Studies: McMaster University School of Nursing.

National Aboriginal Health Strategy Working Party (NAHS. (1989). A National Aboriginal Health Strategy. Canberra: AGPS.

Nutbeam, D. (1986). Health promotion glossary. *Health promotion 1*(1).

Otoo, S., Agapitova, N., & Behrens, J. (2009). The capacity development results framework. A strategic and results-oriented approach to learning for capacity development. Washington DC: World Bank Institute. Learning for Development.

Prout, S. (2012). Indigenous wellbeing frameworks in Australia and the quest for quantification. *Social Indicators Research, 109*(2), 317-336. doi: 10.1007/s11205-011-9905-7

Ritchie, J., & Spencer, L. (1994). Qualitative data analysis for applied policy research. In B. A. & R. Burgess (Eds.), *Analysing qualitative data* (pp. 173-194). London: Routledge.

Sanson-Fisher, R. W., Campbell, E. M., Perkins, J. J., Blunden, S. V., & Davis, B. B. (2006). Indigenous health research: a critical review of outputs over time. *Australasian Medical Publishing Company, 184*(10), 502-505.

Wise, M., Angus, S., Harris, E., & Parker, S. (2012). Scoping study of health promotion tools for Aboriginal and Torres Strait Islander people. Melbourne: Lowitja Institute.
